# Preoperative Metabolic Risk Factors and Outcomes in Living Donor Liver Transplant in HBV Recipients

**DOI:** 10.3390/jcm15020811

**Published:** 2026-01-19

**Authors:** Safiye Koçulu Demir, Ayfer Serin, Birkan Bozkurt, Ender Anılır, Yaman Tokat

**Affiliations:** 1 Department of Infectious Diseases and Clinical Microbiology, Sancaktepe Şehit Prof. Dr. İlhan Varank Training and Research Hospital, 34785 Istanbul, Turkey; safiyekoculu@gmail.com; 2 Department of Gastroenterology, Bağcılar Training and Research Hospital, University of Health Sciences, 34200 Istanbul, Turkey; 3 Department of General Surgery, Başakşehir Çam and Sakura City Hospital, 34887 Istanbul, Turkey; birkan.bozkurt@gmail.com; 4 Department of General Surgery, Ödemiş State Hospital, 35750 İzmir, Turkey; 5 Department of General Surgery, Acıbadem Health Group, 34394 Istanbul, Turkey; yaman.tokat@acibadem.com

**Keywords:** HBV, obesity, liver transplantation, diabetes mellitus, cirrhosis

## Abstract

**Objective:** Additional preoperative risk factors may influence the prognosis of patients diagnosed with HBV. This study aims to compare the effects of cirrhosis patients with HBV with and without risk factors on post-transplant follow-ups and postoperative complications. **Materials and Method:** The study included patients with HBV who underwent living donor liver transplantation (LDLT) at Demiroğlu Bilim University, Şişli Liver Transplant Center, Istanbul, Türkiye, between 2004 and 2019. The data from 319 patients were retrospectively analyzed. Those without preoperative risk factors were classified as group 1 (*n* = 214), and those with risk factors were classified as group 2 (*n* = 105). These patients were compared in terms of complications during their postoperative follow-up. The Student’s *t*-test, ANOVA test, Mann–Whitney U test, Kruskal–Wallis test, chi-squared test, and Fisher’s exact test were used, and *p* < 0.05 was considered statistically significant. **Results:** When group 1 and group 2 were compared in terms of postoperative mortality, infections, bleeding complications, and biliary system complications, no statistically significant difference was found [(8.87% vs. 9.52% *p* = 0.62), (28.80% vs. 20.24%, *p* = 0.95), (6.10% vs. 8.70%, *p* = 0.35), (12.7% vs. 9.60% *p* = 0.19, respectively)]. Although bleeding complications were numerically found more frequent in patients with obesity, this difference did not reach statistical significance (23.02% vs. 6.10% *p* = 0.08). **Conclusions:** Obesity was not significantly associated with postoperative complications and may be influenced by accompanying comorbidities.

## 1. Introduction

Hepatitis B virus (HBV) infection remains a global public health problem with changing epidemiology due to various factors, including vaccination policies and migration [1]. Cirrhosis is a disease characterized by the progression of fibrosis and nodule formation, which develops based on chronic inflammation in the liver [2]. Viral infections, toxins, nonalcoholic steatohepatitis (NASH), and hereditary and autoimmune diseases cause chronic inflammation of the liver, leading to prolonged tissue damage, fibrosis, and cirrhosis. Metabolic dysfunction-associated fatty liver disease (MAFLD) is considered the liver manifestation of metabolic syndrome. MAFLD includes a wide spectrum of liver injuries including simple steatosis and NASH that may lead to serious complications such as liver cirrhosis and hepatocellular carcinoma (HCC) [2,3].

While alcohol addiction and hepatitis C virus (HCV) cause 50% of cirrhosis in developed countries, the main cause in Turkey is HBV and HCV infections [4]. It is estimated that more than 1 million people die every year in the world due to complications such as cirrhosis and liver cancer due to viral hepatitis. It is reported that HBV infection is responsible for 30–40% of cirrhosis cases and 40–50% of liver cancer. The treatment of cirrhosis and HCC is a liver transplantation (LT). Viral hepatitis constitutes more than half of our country’s LT cases performed between 2012 and 2016 [5]. Comorbidities such as obesity, overweight, diabetes mellitus (DM), coronary artery disease (CAD), and insulin resistance in the preoperative period in patients undergoing LT have known risk factors [6]. Metabolic side effects such as weight gain and diabetes may occur in liver recipients due to drug use [7]. The global prevalence of MAFLD is estimated to be 32.4%, with a higher prevalence in males (39.7%) than in females (25.6%) [8]. In recent years, there has been an increase in other medical consequences of metabolic syndrome, including NASH [9,10,11]. In total, 10–15% of NASH patients continue to have inflammation and fibrosis, eventually leading to cirrhosis and HCC. A significant proportion of obese individuals also have a risk of developing hepatocellular carcinoma [12]. Recent global data estimate the prevalence of MAFLD at 32.4%, with a higher rate in men (39.7%) than women (25.6%) [8]. These findings underscore the growing burden of metabolic syndrome-related liver disease. Previous studies have demonstrated that metabolic comorbidities contribute to increased perioperative risks, but data specifically addressing HBV-related cirrhosis remain limited. Therefore, this study aimed to evaluate the impact of preoperative metabolic and cardiovascular comorbidities, namely, DM, HT, HL, CAD, and obesity, on postoperative outcomes, including infection, bleeding, biliary complications, mortality, and length of hospital stay in patients undergoing LT for HBV-related cirrhosis.

## 2. Materials and Methods

### 2.1. Study Design and Population

This retrospective single-center study included adult patients (≥18 years) who underwent living donor liver transplantation (LDLT) for HBV-related liver disease between January 2004 and December 2019 at our tertiary referral center. Preoperative patient data were obtained from records prior to the operation. Patients’ post-transplant follow-up records were reviewed, and 180 months of follow-up were reviewed from the records.

Laboratory parameters were measured at the time of hospital admission and at monthly intervals until liver transplantation. The study used the patient’s most recent preoperative measurement. Donors were selected from healthy relatives aged 20–40 years who had received ethical committee approval.

A total of 1010 liver transplants were performed due to end-stage liver disease caused by various etiological factors, of which 468 were due to HBV-related liver disease. Patients with HBV-related liver disease who underwent living donor liver transplantation were included in the study. Deceased donor liver allotransplant recipients (*n* = 40) were excluded because this study included data from living donor recipient cohorts.

After excluding 109 patients with incomplete medical records, 319 patients were included in the final analysis (Schema 1).

Flowchart

Total LT patients: 1010

        ↓

HBV-related transplants: 468 → (*n* = 40 deceased donors LT)

        ↓

*n* = 428

        ↓

Excluded (missing data): *n* = 109

        ↓

Included in analysis *n*= 319 (living donors LT)

→ Group 1 (*n* = 214, no risk factors)

→ Group 2 (*n* = 105, ≥1 risk factors)

### 2.2. Grouping and Definitions

Patients were classified into two groups based on the presence or absence of preoperative metabolic or cardiovascular risk factors:

Group 1 (*n* = 214): Patients without any preoperative risk factors.

Group 2 (*n* = 105): Patients with at least one preoperative risk factor, including DM, HT, HL, CAD, and/or obesity.

The diagnosis of diabetes mellitus was based on fasting blood glucose ≥ 126 mmg/dL or HbA1c ≥ 6.5 or 2-h PG ≥ 200 mg/dL (≥11.1 mmol/L) during an oral glucose tolerance test (OGTT). The test should be performed as described by the WHO, using glucose load containing the equivalent of 75 g anhydrous glucose dissolved in water. In an individual with classic symptoms of hyperglycemia or hyperglycemic crisis, a random plasma glucose ≥ 200 mg/dL (≥11.1 mmol/L) was used. Random refers to any time of day without regard to the time since the previous meal.

HT on systolic/diastolic blood pressure ≥ 140/90 mmHg or use of antihypertensive medication.

HL on serum LDL cholesterol ≥ 130 mg/d Lor current lipid-lowering therapy.

Obesity was defined as a body mass index (BMI) ≥ 30 kg/m^2^; overweight was defined as a BMI up to 29.9 kg/m^2^.

Coronary artery disease (CAD) was identified based on documented coronary angiography or prior ischemic heart disease.

In this study, we aimed to compare comorbid diseases such as diabetes mellitus (DM), HT, HL, CAD, and obesity in the pre-transplant period in patients who underwent LT due to cirrhosis caused by HBV and HCC with the relationship between these patients’ duration of hospital stay and postoperative complications (postoperative infection, bleeding, reoperation, biliary complications, mortality).

Patient data were obtained from the hospital’s automation system and patient files and scanned retrospectively. Demographic characteristics (age, gender, height, weight, comorbidity, BMI) and the results of laboratory tests including complete blood count, BUN, creatinine, AST, ALT, ALP, GGT, albumin, total bilirubin, direct bilirubin, prothrombin time, INR, TSH, free T3, free T4, AFP, Ca19-9, fasting blood glucose, HbA1c, fasting insulin, HOMA IR, Na, K were reported. For hepatitis B serology, HbsAg, anti-HBs, anti-Hbe, anti-HBc, anti-HDV, HBV DNA, HDV RNA tests were examined. PAP (pulmonary artery pressure), MELD, and CHILD scores were recorded.

Those with preoperative risk factors such as DM, HL, CAD, and obesity were divided into group 2, and those without risk factors were divided into group 1. The groups were compared with each other in terms of complications in the postoperative follow-up, infection, biliary system stenosis or leakage, and mortality rate.

The study was conducted in accordance with the Declaration of Helsinki.

Ethical approval was obtained prior to the initiation of the study (Demiroğlu Bilim University, Istanbul, Turkey 21.072020/13 February 2020).

### 2.3. Statistical Analysis

All data were analyzed using the Statistical Package for the Social Sciences (SPSS-14) Inc., Chicago, IL, USA. Groups were compared statistically with the Student’s *t*-test, ANOVA test, Mann–Whitney U test, and Kruskal test. The chi-squared test and Fisher’s exact test were used for categorical variables. Parametric data were given as mean ± standard deviation, and nonparametric data were given as percentages. A value of *p* < 0.05 was considered statistically significant.

## 3. Results

Three hundred nineteen patients who underwent liver transplantation due to HBV-induced liver cirrhosis were included in the study. 319 (251 male, 78%) patients were divided into two groups according to whether there were risk factors or not. Group 1 included 214 patients without risk factors for the preoperative period, and group 2 included 105 patients with at least one or more risk factors. A comparison of patients’ demographic data between groups is shown in Table 1. There were 165 males (77%) in group 1 and 86 male patients (81%) in group 2, and the difference was not statistically significant (*p* = 0.35).

The mean age was 51.14 ± 9.74 years in group 1 and 51.69 ± 7.58 years in group 2, and there was no statistically significant difference between both groups (*p* = 0.61). There was no significant difference in BMI between the two groups (27.67 ± 5.00 in group 1 and 27.22 ± 4.07 in group 2, *p* = 0.61). The length of hospital stay was 18.69 ± 10.68 days in group 1 and 18.31 ± 10.15 days in group 2 (*p* = 062). There was no statistically significant difference between the two groups in time to death [(6.40 ± 13.37 months vs. 6.00 ±7.07 months *p* = 0.62)] (Table 1). All patients were HBsAg-positive. The mean HBV DNA was 5.18 × 10^6^ ± 5.6 × 10^6^ IU/mL in Group 1 and 5.27 × 10^6^ ± 4.7 × 10^6^ IU/mL in Group 2, and there was no statistical difference between the two groups (*p* = 0.16). When the comparison was made in terms of the MELD-Na score and Child score, no significance was found [(15.79 ± 6.75 vs. 15.07 ± 3.35 *p* = 0.37), (8.23 ± 2.26 vs. 8.09 ± 2.26 *p* = 0.75)], respectively. When the biochemical parameters were evaluated, no statistically significant difference was found between the groups (Table 2).

The patients in group 2 were 105, and the distribution of risk factors is shown in Table 3. While 72 (69.23%) patients had one preoperative risk factor, 30 (28.86%) patients had 2 comorbid diseases, and 2 (1.91%) patients had three comorbid diseases.

A statistical comparison of preoperative risk factors and postoperative complications is shown in Table 4.

There was no statistical difference between group 1 and group 2 in terms of postoperative infections, significant bleeding complications, biliary system complications, strictures, or leaks.

When group 1 and group 2 were compared in terms of postoperative mortality, infections, bleeding complications, and biliary system complications, no statistically significant difference was found [(8.87% vs. 9.52% *p* = 0.62), (28.80%, vs. 20.24%, *p* = 0.95), (6.10% vs. 8.70%, *p* = 0.35), (12.7% vs. 9.60% *p* = 0.19, respectively)]. Although bleeding complications were numerically more frequent in patients with obesity, this difference did not reach statistical significance (23.02% vs. 6.10% *p* = 0.08) (Table 4).

## 4. Discussion

There are studies showing that patients develop obesity, diabetes, and hyperlipidemia after LT. This has been reported to be related to the medications used [7]. In cirrhosis patients, many comorbid diseases such as DM, CAD, HL, and obesity can accompany the disease that affects the prognosis. LT is the curative treatment of cirrhosis. Patients with cirrhosis are prepared for operations by evaluating perioperative risks according to Child and MELD scores. Even cirrhosis alone poses a perioperative risk [13]. There are studies showing that intraoperative hypoxia rates in advanced-stage cirrhosis patients with ascites are between 5 and 32% [14]. In a retrospective study by Teh et al., including 772 patients, perioperative risks were evaluated with the MELD score in patients with cirrhosis. In terms of MELD score, postoperative mortality rates for those with MELD scores < 7, MELD scores = 8–11, and MELD score > 11 are 5.7%, 10.3%, and 25%, respectively [15]. Bleeding, infections, biliary system stenosis, or leaks can be seen in the early and late periods after LT, and these cause an increase in mortality. F. Baganate et al. found that in 67,977 LT patients, the early (>90 days) mortality rate was 5% and the mortality rate within the first year was 10% [16]. In our study, the 1-year mortality was found to be 9.52% in the group with risk factors and 8.87% in the group without risk factors, and no significant difference was found between the groups.

Although the study covered a period of 180 months, long-term results not being available due to reasons such as some patients going abroad and changes in follow-up centers is a limiting factor.

Our study suggests that it may be possible to predict postoperative complications (mortality, length of hospital stay) among patients with and without preoperative risk factors after LT in patients with HBV-related cirrhosis.

Post-LT biliary complications (leakage, stenosis, bilioma) are an important cause of morbidity and mortality [17,18]. The rate of biliary stenosis is 28–32% in living donor transplantation [19]. In our study, post-transplant biliary complications, biliary strictures, biliary leak, and bilioma were considered in total. There was no significant difference between group 1 (12.7%) and group 2 (9.60%) in terms of biliary complications. The biliary complications we detected in our study are similar to those in previous studies [1,5]. Postoperative bleeding is usually seen in the early period and requires urgent intervention. Jung JW et al. found 9% intra-abdominal bleeding among a group of 1039 liver transplant patients (*n* = 94) [20]. In our study, postoperative bleeding (intra-abdominal) complication rates were similar in both groups and were not found to be statistically significant.

Post-transplant infections are an important cause of mortality and morbidity in the first year after the operation. It is 80% during the year, with 70% bacterial infections, 20% viral infections, and 8% fungal infections [21].

The study is limited by the small number of patients with three preoperative risk factors. Despite this small number, it is noteworthy that infection was observed in this group. Future studies with larger numbers of patients in this group are needed.

The present study evaluated the impact of preoperative comorbidities on postoperative outcomes in patients with hepatitis B virus (HBV)-related cirrhosis undergoing liver transplantation (LT). Post-transplant metabolic complications, including obesity, diabetes mellitus (DM), and hyperlipidemia (HL), are well-documented and are often attributed to the use of long-term immunosuppressive agents such as corticosteroids and calcineurin inhibitors [7]. However, the contribution of preexisting metabolic risk factors to postoperative morbidity and mortality has received less attention. Our findings indicate that the overall one-year mortality was similar between patients with and without preoperative risk factors.

The preoperative assessment of liver transplant candidates remains one of the most critical steps in optimizing post-transplant outcomes. The Child–Pugh and Model for End-Stage Liver Disease (MELD) scores are widely used to evaluate the severity of liver disease and predict short-term mortality [13]. Patients with advanced cirrhosis often present with hemodynamic instability, malnutrition, and sarcopenia, all of which contribute to higher perioperative risk. Teh et al. reported that increasing MELD scores were significantly associated with perioperative mortality, with rates of 5.7%, 10.3%, and 25% in patients with MELD < 7, 8–11, and >11, respectively [15].

In this study, early and late postoperative complications such as bleeding, infection, and biliary tract complications were evaluated. Consistent with Baganate et al., who reported early (<90 days) and one-year mortality rates of 5% and 10%, respectively, among 67,977 LT recipients [16], our one-year mortality rate was 7–8%, showing a comparable outcome profile.

The rate of biliary complications in our cohort (8%) was within the range reported [5]: 15% in deceased donors and 28–32% in living donor transplants [17,18,19].

Postoperative bleeding occurred at a rate similar to previous studies, such as the 9% incidence reported by Jung et al. in a cohort of 1039 LT patients [20].

Infections remain a leading cause of morbidity and mortality after LT, particularly in the first postoperative year. Up to 80% of recipients develop at least one infectious episode, with bacterial infections being the most common [21].

Von Son et al. demonstrated that obesity doubled the risk of mortality after LT compared with normal-weight patients [22]. Our findings corroborate this observation and emphasize the need for pre-transplant optimization of metabolic status.

Beyond the direct physiological effects, obesity and associated metabolic syndrome may also influence immunosuppressive drug pharmacokinetics, leading to suboptimal drug exposure or toxicity. Furthermore, the inflammatory milieu characteristic of obesity could exacerbate ischemia–reperfusion injury and impair graft regeneration, resulting in poorer long-term outcomes.

Therefore, integrating multidisciplinary prehabilitation programs including weight management, dietary interventions, and physical conditioning into transplant evaluation may mitigate these risks and enhance postoperative recovery.

This study evaluated the impact of preoperative metabolic and cardiovascular comorbidities on postoperative outcomes among patients with HBV-related cirrhosis undergoing liver transplantation. The main findings can be summarized as follows: the presence of preoperative risk factors such as DM, HT, HL, CAD, or obesity did not significantly affect overall postoperative mortality or biliary complications. However, these findings highlight the importance of comprehensive metabolic and cardiovascular evaluation before LT, even in patients whose liver-specific prognostic scores (e.g., MELD, Child–Pugh) are comparable. Post-transplant metabolic complications, including obesity, diabetes, and hyperlipidemia, have been widely reported and are often attributed to long-term immunosuppressive therapy [7]. In our study, we focused on preexisting comorbidities, demonstrating that their influence on postoperative outcomes begins even before exposure to post-transplant medications.

Given the relatively long timeframe for this study, a comparison of antiviral medications and HBV immunoglobulins used in both pre- and post-transplant hepatitis B management was not conducted. This could be a potential research topic. Timeframe alone may be a confounding factor in the results because selection practices and post-transplant management practices change, and outcomes tend to improve as more experience with living donor liver transplantation is gained.

Similarly, Baganate et al. analyzed over 67,000 LT recipients and reported early (<90 days) and 1-year mortality rates of 5% and 10%, respectively [16]. The mortality rates in our cohort (8.83–9.26%) were comparable to these values.

Van Son et al. showed that obesity doubles the risk of mortality after LT compared with normal-weight recipients [22].

There is also D. Amara’s study; complications were observed in 38% and 47% of 1684 deceased donors and 109 living donor liver transplant cases included from 29 centers, respectively. The most common complications included biliary tract complications (19% deceased donor liver transplant; 31% living donor liver transplant), bleeding requiring reoperation (14% deceased donor liver transplant; 9% living donor liver transplant), and vascular complications (6% deceased donor liver transplant; 9% living donor liver transplant [23]). Some complications were also observed in our study, but our study differs in that it is from a single center and only includes living donor liver transplant patients.

Furthermore, our study differs from previous studies because it compares HBV patients and the relationship between preoperative metabolic risk factors in these patients and post-transplant complications.

The overall mortality did not differ significantly between the two main groups (with and without risk factors). Although bleeding complications were numerically more frequent in patients with obesity, this difference did not reach statistical significance (23.02% vs. 6.10% *p* = 0.08).

The association between metabolic comorbidities can be explained by several mechanisms:

Obesity promotes chronic low-grade inflammation and endothelial dysfunction, increasing susceptibility to infection and impaired wound healing. DM alters immune responses and microcirculation, predisposing patients to bacterial infections and delayed tissue repair. CAD and vascular stiffness may reduce hepatic graft perfusion during and after surgery, affecting graft regeneration. The combination of these conditions amplifies systemic oxidative stress, which can worsen ischemia–reperfusion injury during transplantation. Additionally, obesity may alter the pharmacokinetics of immunosuppressive agents, leading to variable drug exposure and increased risk of toxicity under immunosuppression.

This study contributes to the growing body of literature emphasizing the importance of metabolic health in transplant outcomes. Although our sample size was limited, the consistent trend of higher complication and mortality rates among obese and multi-morbid patients suggests a clinically meaningful relationship. Future multicenter prospective studies with larger cohorts and mechanistic analyses are warranted to further clarify the underlying pathophysiology and to establish evidence-based preoperative optimization strategies. Middleton F and colleagues reported that there are small but real risks for living liver donors [24]. Our study did not provide information about the donors. However, our hospital records include preoperative and postoperative records and follow-ups of the donors. Further studies are needed on this subject. This study investigated the relationship between metabolic risk factors and postoperative complications in HBV liver recipients. This aspect limits the study.

Our findings emphasize the need to integrate metabolic optimization into pre-transplant evaluation. Prehabilitation programs involving dietary counseling, physical conditioning, and glycemic and lipid control may improve both short- and long-term outcomes. Moreover, incorporating comorbidity-based risk indices alongside traditional liver scores (MELD, Child–Pugh) could enhance prognostic accuracy. For instance, including BMI, HbA1c, and cardiovascular parameters in preoperative assessments may help stratify patients more effectively and guide postoperative monitoring. Traditional liver specific risk models, such as MELD and Child–Pugh scores, remain essential for assessing disease severity; however, incorporating metabolic and cardiovascular parameters into preoperative evaluation could enhance prognostic accuracy.

Targeted pre-transplant interventions including weight reduction, glycemic control, lipid management, and cardiovascular risk optimization may reduce postoperative morbidity and improve long-term survival. Future research should focus on developing standardized metabolic optimization protocols prior to transplantation and integrating these strategies into routine pre-transplant assessment.

The main point to remember is that being overweight and having several health problems both raise the risk. The clinical recommendation is that the metabolic profile must be enhanced before liver transplantation. The criteria for validation in prospective research include multicenter studies.

## 5. Conclusions

Preoperative obesity and metabolic comorbidities influence postoperative outcomes after liver transplantation in patients with HBV-related cirrhosis. While traditional liver disease severity scores such as MELD remain essential for risk stratification, the inclusion of metabolic and cardiovascular parameters may improve prognostic accuracy.

Targeted pre-transplant interventions, including weight reduction, glycemic control, and cardiovascular risk optimization, could play a pivotal role in improving survival and reducing postoperative complications.

Future research should focus on developing standardized protocols for metabolic optimization prior to transplantation and integrating lifestyle and pharmacologic approaches to ensure long-term graft and patient survival.

## Figures and Tables

**Table 1:** Comparison of demographic characteristics between groups.

Parameter	Group 1 (*n* = 214) mean ± SD	Group 2 (*n* = 105) mean ± SD	*p* Value
Age (years)	51.14 ± 9.74	51.69 ± 7.58	0.61
BMI (kg/m^2^)	27.67 ± 5.00	27.22 ± 4.06	0.53
MELD score	15.79 ± 6.75	15.07 ± 3.35	0.37
CHILD score	8.23 ± 2.26	8.09 ± 2.27	0.75

Abbreviation: BMI = Body Mass Index, MELD = Model for End-Stage Liver Disease, CHILD = Child–Pugh–Turcotte score. Student’s *t*-test, ANOVA test, Mann–Whitney U test, Kruskal–Wallis test, chi-squared test, and Fisher’s exact test were used, and *p* < 0.05 was considered statistically significant.

**Table 2:** Laboratory parameters and comparison between groups.

Parameter	Group 1 (*n* = 214) Mean ± SD	Group 2 (*n* = 105) Mean ± SD	*p* Value
Hb (g/dL)	12.40 ± 3.12	13.06 ± 4.36	0.13
Htc (%)	34.94 ± 6.22	35.92 ± 6.55	0.20
WBC (×10^3^/μL)	3.58 ± 13.12	4.29 ± 15.68	0.67
PLT (/μL)	87,318.78 ± 45,220.66	99,872.54 ± 64,339.30	0.80
INR	1.61 ± 0.49	1.62 ± 0.54	0.897
AST (U/L)	87.44 ± 74.52	87.62 ± 69.08	0.90
ALT (U/L)	60.88 ± 61.92	62.05 ± 38.86	0.86
ALP (U/L)	148.6 ± 95.4	147.6 ± 81.8	0.93
GGT (U/L)	86.7 ± 90.0	101.1 ± 127.1	0.26
Albumin (g/L)	3.17 ± 0.68	3.17 ± 0.67	0.96
Total Bilirubin (μmol/L)	4.52 ± 6.73	4.57 ± 6.57	0.94
Direct Bilirubin (μmol/L)	2.15 ± 2.00	2.16 ± 1.93	0.98
BUN (mg/dL)	24.01 ± 64.57	19.07 ± 13.00	0.44
Creatinine (mg/dL)	0.86 ± 0.33	1.24 ± 3.96	0.17
Na (mEq/L)	135.21 ± 5.52	133.82 ± 14.57	0.23
Glucose (mg/dL)	108.67 ± 39.75	108.77 ± 42.30	0.98
HbA1c (mmol)	5.30 ± 1.20	5.43 ± 1.21	0.42
Insulin (μIU/mL)	20.42 ± 14.03	20.29 ± 15.00	0.95
HOMA-IR (mmol/L)	6.33 ± 9.51	6.70 ± 7.00	0.78
Total Cholesterol (mg/dL)	126.55 ± 45.83	131.47 ± 49.26	0.41
Triglyceride (mg/dL)	78.89 ± 32.27	85.11 ± 36.99	0.15
FT4 (pmol/L)	11.54 ± 12.21	10.31 ± 7.07	0.38
FT3 (pmol/L)	3.53 ± 1.02	3.74 ± 1.37	0.63
TSH (μIU/mL)	1.98 ± 1.76	2.05 ± 1.43	0.70
CEA (ng/mL)	3.96 ± 2.56	5.54 ± 13.48	0.13
CA 19-9 (U/mL)	77.32 ± 201.89	82.06 ± 321.49	0.78
AFP (ng/mL)	51.79 ± 160.70	52.66 ± 149.35	0.39
HBV DNA (IU/mL)	5.18 × 10^6^ ± 5.6 × 10^6^	5.27 × 10^6^ ± 4.7 × 10^6^	0.15

Abbreviations: WBC = White blood cells; Hb = Hemoglobin; Htc = Hematocrit; PLT = Platelet; ALT = Alanine aminotransferase; AST = Aspartate aminotransferase; ALP = Alkaline phosphatase; GGT = Gamma-glutamyl transferase; TSH = Thyroid-stimulating hormone; FT3 = Free triiodothyronine; FT4 = Free thyroxine; HOMA-IR = Homeostatic Model Assessment of Insulin Resistance; HbA1c = Hemoglobin A1c; AFP = Alpha-fetoprotein; HBV DNA = Hepatitis B virus DNA. Student’s *t*-test, ANOVA test, Mann–Whitney U test, Kruskal–Wallis test, chi-squared test, and Fisher’s exact test were used, and *p* < 0.05 was considered statistically significant.

**Table 3:** Distribution of preoperative risk factors in patients with HBV-related liver disease (Group 2, *n* = 105).

Risk Factors	*n* = 105 (%)
Diabetes Mellitus (DM)	29 (27.6)
Chronic Obstructive Pulmonary Disease (COPD)	5 (4.7)
Hypertension (HT)	7 (6.8)
Hyperlipidemia (HL)	3 (2.8)
Coronary Artery Disease (CAD)	16 (15.6)
Obesity	3 (2.8)
Heart Valve Disease (HVD)	9 (8.5)
DM + COPD	6 (5.7)
DM + HT	9 (8.5)
DM + HVD	2 (1.9)
DM + CAD	5 (4.7)
HT + CAD	3 (2.8)
HT + HVD	3 (2.8)
HL + HVD	1 (0.9)
CAD + HVD	2 (1.9)
DM + HT + HL	1 (0.9)
DM + HT + CAD	1 (0.9)

Abbreviations: DM = Diabetes Mellitus; COPD = Chronic Obstructive Pulmonary Disease; HT = Hypertension; HL = Hyperlipidemia; CAD = Coronary Artery Disease; HVD = Heart Valve Disease. Student’s *t*-test, ANOVA test, Mann–Whitney U test, Kruskal–Wallis test, chi-squared test, and Fisher’s exact test were used, and *p* < 0.05 was considered statistically significant.

**Table 4:** Postoperative complications according to Group 1 and Group 2.

Complication	Group 1 (*n* = 214)	Group 2 (*n* = 105)	*p*-Value
Postoperative mortality	8.87%	9.52%	0.62
Infection	28.80%	20.24%	0.95
Bleeding complication	6.10%	8.70%	0.35
Biliary system complication	12.7%	9.60%	0.19
Bleeding (obese vs. non-obese)	23.02%	6.10%	0.08

Student’s *t*-test, ANOVA test, Mann–Whitney U test, Kruskal–Wallis test, chi-squared test, and Fisher’s exact test were used, and *p* < 0.05 was considered statistically significant.

## Data Availability

The original contributions presented in the study are included in the article, further inquiries can be directed to the corresponding authors.
